# Knockdown of five trehalase genes using RNA interference regulates the gene expression of the chitin biosynthesis pathway in *Tribolium castaneum*

**DOI:** 10.1186/s12896-016-0297-2

**Published:** 2016-09-06

**Authors:** Bin Tang, Ping Wei, Lina Zhao, Zuokun Shi, Qida Shen, Mengmeng Yang, Guoqiang Xie, Shigui Wang

**Affiliations:** Hangzhou Key Laboratory of Animal Adaptation and Evolution, College of Life and Environmental Sciences, Hangzhou Normal University, Hangzhou, Zhejiang China

**Keywords:** *Tribolium castaneum*, Trehalase, RNA interference, Chitin biosynthesis pathway, Gene function

## Abstract

**Background:**

RNA interference is a very effective approach for studies on gene function and may be an efficient method for controlling pests. Trehalase is a key gene in the chitin biosynthesis pathway in insects. Five trehalase genes have been cloned in *Tribolium castaneum*, though it is not known whether the detailed functions of these trehalases can be targeted for pest control.

**Results:**

The functions of all five trehalase genes were studied using RNAi, and the most important results showed that the expression of all 12 genes decreased significantly from 12 to 72 h compared with the control groups, except *GP1* at 72 h, when the expression of the *TcTre2* gene was suppressed. The results also revealed different abnormal phenotypes, and the observed mortality rates ranged from 17 to 42 %. The qRT-PCR results showed that the expression of *TPS*, *GS*, two *GP*, *CHS1a* and *CHS1b* genes decreased significantly, while that of the *CHS2* gene decreased or increased after RNAi after the five trehalases were silenced at 48 h. In addition, *TPS* gene expression decreased from 12 to 72 h after dsTcTre injection.

**Conclusions:**

These results demonstrate that silencing of any individual trehalase gene, especially *Tre1*-*4* and *Tre2* gene can lead to moulting deformities and a high mortality rate through the regulation of gene expression in the chitin biosynthesis pathway and may be a potential approach for pest control in the future.

**Electronic supplementary material:**

The online version of this article (doi:10.1186/s12896-016-0297-2) contains supplementary material, which is available to authorized users.

## Background

RNA interference (RNAi), which is a robust and powerful experimental tool, has been widely used to study gene function through the suppression of gene expression [1]. Many reports on the use of double-stranded RNA (dsRNA) techniques to silence target genes that are resistant to insecticides have indicated that RNAi may be an efficient method for controlling insect pests, and novel pest management strategies involving the modification of plants are being developed [2–4]. In addition, RNAi technology has been widely used to investigate the functions of essential genes in insects [5]. RNAi has also been employed for the silencing of vital genes in *Tribolium castaneum*, *Nilaparvata lugens* and other insects [4, 6]. Furthermore, RNAi could be used in novel methods of pest management and may be more effective than other pest control measures once the problems of the efficiency and specificity of gene silencing are solved [7].

The chitin biosynthesis pathway is crucially important for insect life; the pathway regulates the main component of the cuticle of most insects and the peritrophic membranes of arthropods [8–13]. The first enzyme involved in the chitin biosynthesis pathway is trehalase (Tre, TRE or Treh), which hydrolyses trehalose into glucose as a source of the substrate for hexokinase (HK), while the last enzyme in the pathway is chitin synthase (CHS), which consists of two classes, CHS1 and CHS2, in the majority of insects [14, 15]. Glycogen phosphorylase (GP) in the decomposition pathway together with glycogen synthase (GS) in the synthetic pathway regulates glycogen levels. Glucose-6-phosphate generated through the hydrolysis of trehalose and degradation of glycogen is transformed into fructose-6-phosphate by glucose-6-phosphate isomerase (GPI), which plays different roles in the chitin biosynthesis pathway after the enzymatic action of glutamine: fructose-6-phosphate transaminase (GFAT) and in the glycolysis pathway after the enzymolysis of phosphofructokinase (PFK) (Additional file 1: Figure S1) [16–20].

In *T. castaneum*, there is one trehalose 6-phosphate synthase (TPS) that catalyses the synthesis of a trehalose from two glucose molecules, in addition to HKs (HK1 and HK2), one glycogen synthase (GS), two glycogen phosphorylases (GP1 and GP2), one glucose-6-phosphate isomerase (GPI), one fructose-6-phosphate transaminase (GFAT) and one phosphofructokinase (PFK). The two classes of chitin synthase (CHS1 and CHS2) are found in *T. castaneum*, and the CHS1 yields two alternatively spliced transcripts and the corresponding isoforms of the enzyme proteins, namely CHS1a and CHS1b [9]. CHS1 is responsible for moulting during the entire lifecycle of *T. castaneum*, and CHS2 is required for the formation of chitin in the peritrophic membrane (PM) and RNAi of this gene leads to reduced food uptake, growth arrest and failure to molt to the next instar insect. In addition, CHS1a is responsible for both the larval-pupal and pupal-adult moults, while CHS1b is only responsible for the latter [9].

Trehalase (a-glucoside-1-glucohydrolase, EC 3.2.1.28) is an anomer-inverting α-trehalose-1-D-glucosidase that hydrolyses a trehalose molecule into two glucose molecules. Two forms of trehalase, soluble trehalase (Tre1) and membrane-bound trehalase (Tre2), have been identified to date and cloned in several insect species, including *Tenebrio molitor* (*T. molitor* Coleoptera), *Pimpla hypochondriaca* (Hymenoptera), *Bombyx mori* (*B. mori* Lepidoptera), *Apis mellifera* (Hymenoptera), and *Spodoptera exigua* (*S. exigua* Lepidoptera) [21–27]. The first soluble and membrane-bound trehalases were found in *T. molitor* and *B. mori*, respectively [21, 23]. Previous research has shown that two types of trehalases regulate different CHS enzymes [28]. It has been reported that trehalase regulates the expression of chitin synthase in the insect cuticle and midgut, and inhibiting chitin biosynthesis kills pests when trehalase gene expression is suppressed or knocked down [28, 29]. However, the functions and relationships of many TcTres and whether and how they regulate different CHS enzymes are unknown, as indicated by Chen and Arakane [9, 28]. Trehalase has become an important target in pest management and control, and new and effective trehalase inhibitor products with practical significance as pesticides are being developed [30].

In the present study, five *T. castaneum* trehalase (*TcTre*) genes were identified and cloned, including four soluble trehalase genes (*TcTre1*-*1*, *TcTre1*-*2*, *TcTre1*-*3* and *TcTre1*-*4*) and one membrane-bound trehalase gene (*TcTre2*); the results indicated that these trehalases may have different functions. The flour beetle, *T. castaneum*, is a member of the coleopteran order of insects. *T. castaneum* is a holometabolous insect in the larva, pupa and adult stages and represents a serious pest that damages grain in storage. It is thought to be an effective and reliable insect for conducting RNAi research [7, 31, 32]. It has also been reported that RNAi is highly effective in *Tribolium*, even in the postembryonic stages of *Tribolium* [9, 33]. Therefore, it will be useful to study the characteristics and functions of the five *TcTre* genes using RNAi. The transcription levels of the five *TcTre* genes were found to be substantially down-regulated after specific dsRNA injection targeting every *TcTre*. The present study revealed the specific functions of the five *TcTres* genes in chitin biosynthesis in insects as well as some functions that are common to all of them.

## Methods

### Insect cultures

The *T. castaneum* insects were kindly provided by Professor Chao-Ming Wei of Shanxi Normal University. The experimental beetle populations were raised in whole wheat flour with 5 % yeast powder at 30 ± 1 °C [7, 34]. The developmental stages were synchronised by providing fresh coarse wheat bran to replace the old coarse wheat bran every day, and the old coarse wheat bran mixture containing *T. castaneum* eggs was cultured for future experiments.

### RNA extraction, cDNA synthesis and rapid amplification of full-length cDNA (PCR)

Total RNA was extracted from the whole body of *T. castaneum* larvae or adults using the TRIzol (Invitrogen, Carlsbad, California, USA) method. The RNA concentration was then determined by measuring the absorbance at 260 nm with a spectrophotometer [35]. The purified RNA was stored at -80 °C for future experiments. First-strand cDNA synthesis was performed using the PrimeScript® RT reagent Kit with gDNA Eraser (Takara). The obtained first-strand cDNA (1 μl) was used as the template for PCR.

### Cloning of the fragments of five trehalase genes with the protein coding regions

Five pairs of specific primers based on the whole gene sequences published in GenBank were designed for *TcTre1*-*1* cDNA (XM968798), *TcTre1*-*2* cDNA (XM968883), *TcTre1*-*3* cDNA (XM968859), *TcTre1*-*4* cDNA (XM968826) and *TcTre2* cDNA (EFA11183) (Table 1). The components of the PCR mixture included PCR buffer containing 0.1 mM dNTPs, 0.2 μM each primer, and 0.5 U of HiFi-Taq DNA polymerase (Transgene, China) in a total volume of 25 μl. The amplification reactions were performed with the specific primers according to the following conditions: 10 min at 94 °C, followed by 30 cycles of 30 s at 94 °C, 30 s at 55 °C and 120 s at 72 °C, and then 10 min at 72 °C [25].

**Table 1:** Primers used in present study

Gene	Application type	Primer set	Forwad (5’-3’)	Reverse (5’-3’)	Length of target fragment (bp)
*TcTre1*-*1*	cDNA cloning	TcTre1-1	GAACCACTTGTCCCTTGTC	TGCTAATCAAGTGGGCTT	1626
	qRT-PCR	QTcTre1-1	AACGACTCGCAATGGCTGG	CGGAGGCGTAGTGGAATAGAG	127
	dsRNA synthesis	dsTcTre1-1	CGACCTGAAATTAGCCCAGAAG	GGCTCCCCCACTCTTTC	357
*TcTre1*-*2*	cDNA cloning	TcTre1-2	CGTTTCATGTCGTTGTG	GAACACCACAGAGACGTAA	1692
	qRT-PCR	QTcTre1-2	GTGCCCAATGGGTTTATCG	CAACCACAACACTTCCTTCG	261
	dsRNA synthesis	dsTcTre1-2	GGACAATGAGTTGGGGCAG	AACCGCGTAAAATCGCTCG	493
*TcTre1*-*3*	cDNA cloning	TcTre1-3	ATGCCTTTCCTCCTACTTCTCACCC	GGTACTACGGCCGGGAAAAATAA	1647
	qRT-PCR	QTcTre1-3	CCTCTCATTCGTCACAAGCG	AAGCGTTTGATTTCTTTGCG	205
	dsRNA synthesis	dsTcTre1-3	GGGACCGTGGGTTAAACCAAC	CTGTGGCCGCTGAACTGAA	376
*TcTre1*-*4*	cDNA cloning	TcTre1-4	ATGAAGTCTCTCCTCGTTTCG	GATTTTACGCGGTTTCGG	1662
	qRT-PCR	QTcTre1-4	ACGGTGCCCGCATCTACTA	GTGTAGGTGGTCCCGTTCTTG	187
	dsRNA synthesis	dsTcTre1-4	ACGATATCGAGCTCAGAGTCC	TACGTCTCTGTGGTGTTCGC	462
*TcTre2*	cDNA cloning	TcTre2	TTAGTGTGTTGTGCGTTTGT	TGCGAAGAGCACGAGATAA	1929
	qRT-PCR	QTcTre2	CTCAGCCTGGCCCTTAGTTG	GGAGTCCTCGTAGATGCGTT	120
	dsRNA synthesis	dsTcTre2	GGTGCGCTCCAACTACAAAG	GCTCCGCAGTTCCGTGTAG	399
*TcTPS*	qRT-PCR	QTcTPS	CCTTGTTCCACTCAATGCCCGAC	GTCATGTATCCAGATCAAGGGAAC	105
*TcHk1*		QTcHk1	CGCACCGAATGCCAGAATC	GACCCACCCGACATCGATT	141
*TcHk2*		QTcHk2	CGAATCGGCCTAATAGTTGGC	GACGGAGCCCTCGATTTCAT	155
*TcCHS1a*		QTcCHS1a	CGTATAGCCGCCGACTTGAA	CCTATGACGAGAGCACCCAAGA	173
*TcCHS1b*		QTcCHS1b	CCAGGATTGCAAAGGAGTTG	TTGAGGAAACGTCCGAGGTC	179
*TcCHS2*		QTcCHS2	GAGTTGTGGCAATGTTCTCGC	GTGGTGTGGCCCTTGGTT	116
*TcGS*		QTcGS	GGAGTTTCATGCACGGACTG	GTTGGCTTGTCGATGGGAA	105
*TcGP1*		QTcGP1	GCGAGCGACTATGAACTGATG	TTGCGTGTCGGAAACTACAT	104
*TcGP2*		QTcGP2	GGACGAATATTACACAAGTTACGAC	CAGACCCACGATTACGCTT	119
*TcGPI*		QTcGPI	GTGATGCCGGAGGTGAAT	CACGTCGGTGATGGGCTT	112
*TcGFAT*		QTcGFAT	GGAACTGGACATGGACCGTA	GAACGGTGAGGATGCGAGTT	126
*TcPFK*		QTcPFK	GAGCAAGGACATGGAAGGGAA	CCAACCCAACCAGCCACTT	158
*Tcβ*-*actin*		QTcactin	AGGGCGTCATGGTCGGTAT	TTCTACAACGAGCTCCGCG	165
*GFP*	dsRNA synthesis	dsGFP	AAGGGCGAGGAGCTGTTCACCG	CAGCAGGACCATGTGATCGCGC	206

The products were subjected to agarose gel electrophoresis. The true electrophoretic DNA bands corresponding to the expected size of approximately 1600–2000 bp were excised from the agarose gel and purified using a DNA gel extraction kit (OMEGA, USA). The purified DNA was ligated into the pMD18-T vector (TaKaRa, Japan) and sequenced using the dideoxynucleotide method.

### Analysis of the *TcTres* tissue distribution using semi-quantitative PCR

cDNA synthesis and RT-PCR were performed to analyse the tissue distribution of *TcTres* using gene-specific primers [19]. The midgut, fat body, cuticle and Malpighian tubules from mixed larvae including the penultimate larval instar and last-instar larvae were dissected in a saline solution (0.75 % NaCl) and stored at -80 °C for future analysis. Total RNA was isolated from these tissues and reverse transcribed into cDNA. Five pairs of specific primers based on the previously cloned *TcTre* genomic DNAs and one pair of specific of *Tcβ*-*actin* primers were designed (Table 1), which were the same as the primers used in qRT-PCR. First, the content of cDNA in each tissue was adjusted to ensure that the brightness of every electrophoresis band was the same, based on the cloning of *β*-*actin*, and the corresponding amount of cDNA was then added to clone every *TcTre* under the same PCR conditions indicated above to produce specific expression in the four tissues.

### RNAi targeting the five trehalase genes

The cDNA regions of the five *TcTre* genes showing the greatest sequence divergence were targeted for dsRNA production. One region was the target for the *TcTre1*-*1*, *TcTre1*-*2*, *TcTre1*-*3* and *TcTre1*-*4* cDNA regions, as shown in Additional file 1: Figure S2. The lengths of the homologous dsRNAs for *TcTre1*-*1*, *TcTre1*-*2*, *TcTre1*-*3* and *TcTre1*-*4* used in this study were 357, 493, 376 and 462 bp, respectively. The other region was the target for TcTre2, for which the dsRNA contained a unique sequence near the 3’end, and its length was 399 bp (Additional file 1: Figure S2). The highest and lowest nucleotide sequence identities between the sequences were 53.2 and 39.2 %, respectively. Five pairs of primers with the T7 RNA promoter sequence flanking the 5’-ends were designed according to these regions (Table 1), which were synthesized using the Promega T7 Expression kit. Templates for in vitro transcription were prepared via PCR using gene-specific primers with the T7 polymerase promoter sequence at both ends [7].

The NT-88-V3 series (Nikon) micromanipulator system was used. The dorsal side of the first abdominal segment of *T. castaneum* was selected as the injection point, and 0.5–0.7 μg of the dsRNAs (3 μg/μl) targeting the five *TcTres* sequences and *GFP* was injected into each insect [32].

### Sample collection and phenotype observations

After the dsRNAs were injected into last-instar insects, we collected 12 larvae (showing malformation at best) and divided them into three tubes every 12 h for the first 48 h, which were then stored at -80 °C. Most of the larvae developed into the prepupa stage by 48 h after RNAi treatment. The abnormal pupae were collected according to the above method at 72 h after RNAi treatment, when most of the insects had formed pupae. We observed the phenotypes of the insects every 12 h. When the dsRNAs were injected into penultimate-instar insects, samples were collected at 48 and 72 h, and the penultimate-instar insects started to moult to become last-instar insects at 72 h.

### qRT-PCR analyses of *TcTres* and the expression levels of related genes

Total RNA was isolated from *T. castaneum* on each day of the insect’s life cycle, including the larval, pupal and adult stages, and 1 μg of total RNA was extracted and used as the template to demonstrate the stability of *Tcβ*-*actin*. PCR was performed with the *QTcactin*-F and *QTcactin*-R primers under the following conditions: 95 °C for 5 min, followed by 28 cycles of 95 °C for 30 s, 55 °C for 30 s and 72 °C for 30 s, with a final extension at 72 °C for 10 min. The expression of *TcTres* was estimated through qRT-PCR using the Bio-Rad CFX96^TM^ system (Bio-Rad, America) and SsoFast^TM^ EvaGreen Supermix (Bio-Rad, America). All primers were designed to determine the expression of the corresponding homologous gene sequences, which included *TcTre1*-*1*, *TcTre1*-*2*, *TcTre1*-*3*, *TcTre1*-*4*, *TcTre2*, *CHS1a*, *CHS1b*, *CHS2*, *GS*, *GP1*, *GP2*, *HK1*, *HK2*, *GPI*, *GFAT* and *PFK* (Table 1); these primers were designed based on the unique regions of the sequences, which were identified in the alignment using Vector NTI Suite 7. Each reaction was performed in a final volume of 20 μl containing 1 μl of the cDNA sample (or standard), 1 μl (l0 μmol/μl) of each primer, 7 μl of RNAase-free and DNAase-free water and 10 μl of SsoFast^TM^ EvaGreen Supermix. After 3 min of initial denaturation at 95 °C, the cycling protocol consisted of 40 cycles of denaturation at 95 °C for 5 s, annealing at 55 °C -62.5 °C for 20 s and, finally, the melting curve was performed at 65–95 °C (according to the instructions for SsoFast^TM^ EvaGreen Supermix). Standard curves were obtained using ten-fold serial dilutions of pooled total RNA.

### Statistical analyses

The mRNA expression levels in the non-injected group and the dsGFP-injected group were designated as the controls. All of the data obtained in this study are presented as the means ± standard errors (SEs) of 3–6 replicates and were analysed through one-way ANOVA. A *P*-value of less than 0.05 or 0.01 was considered significant or extremelysignificant in Duncan’s new multiple range test (DMRT) [36]. An asterisk indicates a significant difference in mRNA levels between the dsGFP group and each of the dsTcTres-injected groups measured at the same time (p, 0.05, *T* test), and a double asterisk indicates a highly significant difference (p, 0.01, *T* test).

## Results

### Analysis of the tissue distribution of the five *TcTres*

**Fig. 1:**
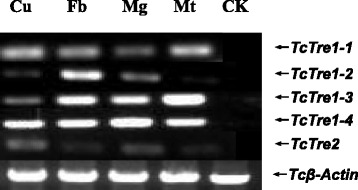
Analysis of the five *TcTre* transcripts in different tissues of last-instar larvae using semi-quantitative PCR (RT-PCR). Total RNA was extracted from various tissues: fat body (Fb), midgut (Mg), cuticle (Cu) and Malpighian tubules (Mt). The expression of *Tcβ*-*actin* in the four tissues was used to standardize the relative expression levels of the five *TcTre* genes. CK represents PCR products without a template used as the controls

The tissue-specific expression of *TcTres* was determined through semi-quantitative reverse transcription PCR (RT-PCR). All five *TcTres* transcripts were detected in the midgut, fat body, cuticle and Malpighian tubules (Fig. 1). *TcTre1*-*1* expression was higher in the cuticle, fat body and Malpighian tubule and lower in the midgut. The highest and lowest *TcTre1*-*2* expression levels were observed in the fat body and Malpighian tubules, respectively. *TcTre1*-*3* expression was higher in the midgut, fat body and Malpighian tubules than in the cuticle. The expression of *TcTre1*-*4* was high in the midgut, fat body, cuticle and Malpighian tubules, whereas that of *TcTre2* was low; the negative control exhibited no expression.

### Efficiency of *TcTre* RNAi in penultimate-instar larvae

**Fig. 2:**
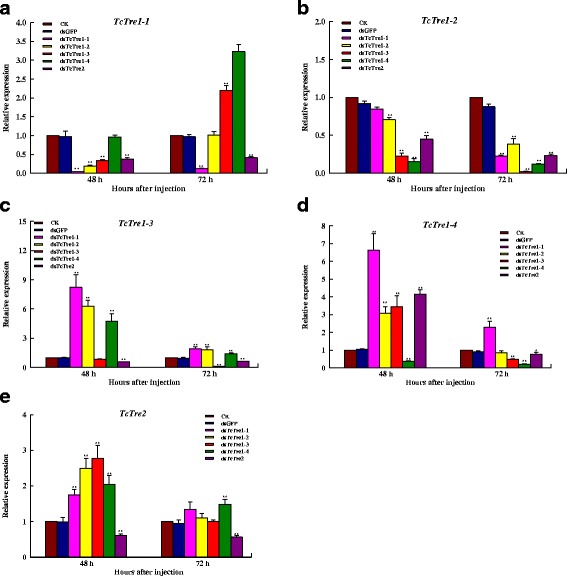
Changes in the mRNA levels of the five *TcTres* after specific RNAi in penultimate-instar larvae. The penultimate-instar larvae were selected as the targets for dsRNA injection. **a**, **b**, **c**, **d** and **e** represent the mRNA levels of *TcTre1*-*1*, *TcTre1*-*2*, *TcTre1*-*3*, *TcTre1*-*4* and *TcTre2* relative to *Tcβ*-*actin* mRNA level after RNAi targeting all five of the *TcTres*, analysed via qRT-PCR

RNAi targeting each *TcTre* was successful in each group, and the expression of the five trehalase genes had decreased significantly at 48 and 72 h after the corresponding dsRNA injections (Fig. 2). In addition, the transcript levels for any of these Tre genes are suppressed not only by the dsRNA for that gene, but also by dsRNA’s for other Tre genes as well (Fig. 2a, b, c and d). All of the soluble *TcTre* transcripts decreased significantly after dsTcTre2 injection, except for *TcTre1*-*4* at 48 h. *TcTre2* transcript levels increased after RNAi targeting all of the soluble dsTcTres (Fig. 2e). Following RNAi treatment, most of the penultimate-instar larvae exhibited sloughing and became last-instar larvae at 72 h.

### Efficiency and specificity of RNAi targeting *TcTres* in last-instar larvae

**Fig. 3:**
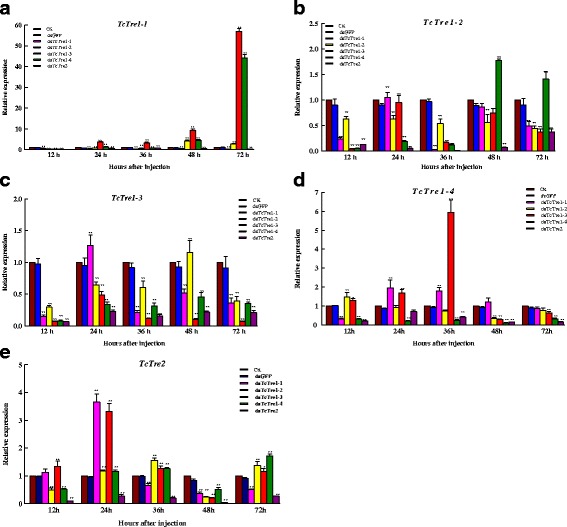
Changes in the mRNA transcripts of the five *TcTre* genes after specific RNAi in last-instar larvae. **a**, **b**, **c**, **d** and **e** represent the mRNA levels of *TcTre1*-*1*, *TcTre1*-*2*, *TcTre1*-*3*, *TcTre1*-*4* and *TcTre2* relative to the *Tcβ*-*actin* mRNA level after RNAi targeting all five of the *TcTres*, analysed via qRT-PCR. Last-instar larvae were chosen as the targets for dsRNA injection

To verify the specificity of RNAi targeting the *TcTre* genes, the alignment of the five dsTcTres was assessed, and no consecutive identical 19-bp sequences between pairs of fragments were found (Additional file 1: Figure S2). All of the *TcTres* decreased significantly from 12 to 72 h after dsRNA injection compared with the control group, and *TcTre2* decreased most significantly after dsTcTre2 RNAi (Fig. 3). The corresponding RNAi efficiencies for *TcTre1*-*1*, *TcTre1*-*2* and *TcTre1*-*3* were 68, 56 and 93 % after 72 h, whereas those of *TcTre1*-*4* and *TcTre2* were 89 and 96 %, respectively, after just 48 h. The efficiencies obtained for *TcTre1*-*3*, *TcTre1*-*4* and *TcTre2* were higher than for the other *TcTres*.

The levels of the different *TcTres* showed both increases and decreases in every *TcTre* RNAi group (Fig. 3), whereas all of the *TcTres* showed decreases in the *TcTre2* RNAi group. Also the transcript level of TcTre1-2 is suppressed not only by the conrresponding dsRNA, but also by other Tre dsRNA’s genes as well (Fig. 3b), the transcript level of *TcTre2* increased after RNAi targeting the other soluble *TcTres*, except after 36 and 72 h in the *TcTre1*-*1* RNAi group and 48 h in all of the soluble *TcTre* RNAi groups (Fig. 3e). The expression of *TcTre1*-*1* increased and decreased after RNAi targeting the other soluble *TcTres*; however, it increased rapidly in the *TcTre1*-*3* RNAi group at 24 h and in the *TcTre1*-*4* RNAi group at 72 h (Fig. 3a). *TcTre1*-*2* only showed an obvious increased at 48 and 72 h after *TcTre1*-*4* RNAi (Fig. 3b). *TcTre1*-*3* increased slightly at 24 h after *TcTre1*-*1* RNAi and at 48 h after *TcTre1*-*2* RNAi, although a significant reduction was detected in the other groups (Fig. 3c). There was variation in the expression of *TcTre1*-*4* in the other soluble RNAi groups, and it peaked at 36 h in the *TcTre1*-*3* RNAi group (Fig. 3d).

### Analyses of phenotypes, aberrations and survival rates after RNAi

**Fig. 4:**
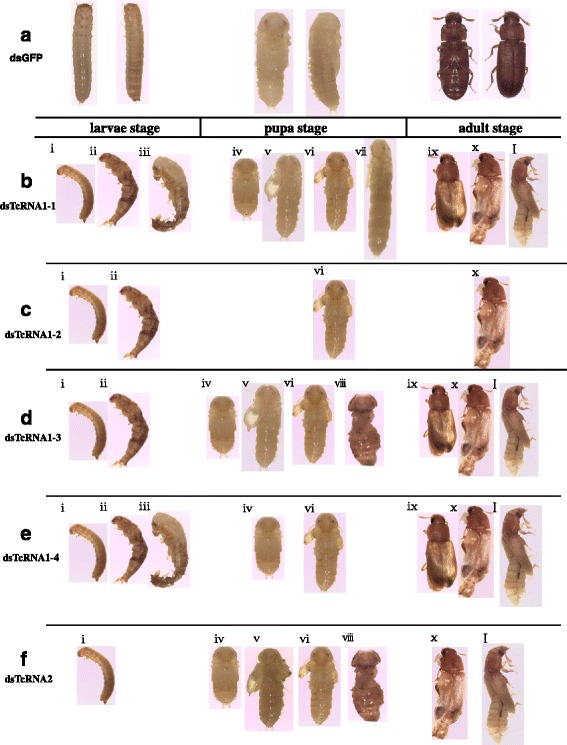
Abnormal phenotypes were caused by RNAi targeting the five dsTcTres. All abnormal insects in the three stages (larva-pupa stage, pupal stage, and adult stage) are displayed and are divided into eleven types, from i to xi, after RNAi. **a** dsGFP-injected group. **b** dsTcTre1-1-injected group. **c** dsTcTre1-2-injected group. **d** dsTcTre1-3-injected group. **e** dsTcTre1-4-injected group. **f** dsTcTre2-injected group. The percentage below each type of abnormal insect is the corresponding proportion among all of the abnormal insects in that stage, such as the larva-pupa stage

With the successful silencing of every *TcTre*, the insects subjected to RNAi exhibited a large number of deaths and presented various abnormal phenotypes (Fig. 4). The mortality rates recorded after RNAi targeting the five *TcTres* were as follows: *TcTre1*-*1* (35 %), *TcTre1*-*2* (17 %), *TcTre1*-*3* (23 %), *TcTre1*-*4* (42 %) and *TcTre2* (40 %) (Table 2). These values corresponded to 35, 12, 18, 35 and 28 insects and were higher than the death rates of the naive insects (0 %) and the insects injected with dsGFP (7 %). The death rates differed in different insect stages. The following percentages were obtained for the number of deaths observed among every type of abnormal insect divided by the total number of abnormal insects in the same stage (larva, pupa or adult stages). From larva to pupa, the percentages were *TcTre1*-*1* (20 %), *TcTre1*-*2* (14 %), *TcTre1*-*3* (13 %), *TcTre1*-*4* (24 %) and *TcTre2* (8 %), which corresponded to 20, 11, 10, 24 and 6 larvae that died before the pupal stage. During the pupa-adult stage, the percentages were *TcTre1*-*1* (8 %), *TcTre1*-*2* (0), *TcTre1*-*3* (4 %), *TcTre1*-*4* (13 %) and *TcTre2* (24 %), which corresponded to 6, 0, 3, 7 and 16 pupae that died before becoming adults, and most of these numbers were higher than in the two control groups. There were some deaths recorded during the adult stage, when the following percentages were determined: *TcTre1*-*1* (12 %), *TcTre1*-*2* (2 %), *TcTre1*-*3* (8 %), *TcTre1*-*4* (6 %) and *TcTre2* (12 %), which corresponded to just 9, 2, 5, 3 and 6 more adults than in the two control groups. Additional details are presented in Table 2, including the aberration rates, survival rates, pupation rates and eclosion rates (Table 2).

**Table 2:** *Tribolium castaneum* phenotype rate after RNAi

dsRNA	aberration rate (%)	survival rate (%)	pupation rate (%)	eclosion rate (%)
dsTcTre1-1	38	65	92	93
dsTcTre1-2	17	83	100	100
dsTcTre1-3	24	77	100	96
dsTcTre1-4	45	58	95	88
dsTcTre2	40	61	100	76
dsGFP	0	94	92	90
no-injection	0	100	100	100

**Fig. 5:**
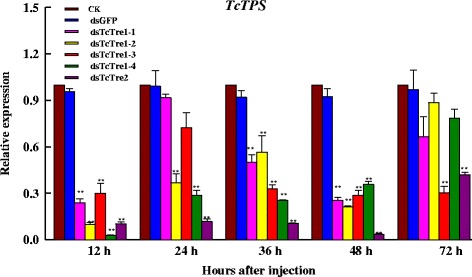
Effects of RNAi targeting the five *TcTres* on trehalose-6-phosphate synthase (TPS) in last-instar larvae. The levels of *TPS* mRNA relative to the *Tcβ*-*actin* mRNA level were measured via qRT-PCR

Insects in the larval, pupal and adult stages were present after RNAi targeting dsGFP as well in every *TcTre* RNAi group (Fig. 5). Three abnormal phenotypes were observed in the larval stage, and the insects died with the abnormal phenotype: the body size of the larvae decreased (i), or the size of the larvae abdomen decreased (ii) (both of which were associated with a reduced food intake that mainly occurred from 24 to 36 h after *TcTre* RNAi), and some larvae could not exuviate the larval cuticle to become pupae (iii). During the pupal stage, five malformations were present, which included shrunken pupae that were much smaller than normal pupae (iv); pupae with one set or both sets of wings together with membranous bulging wings (v); pupae with one set or both sets of wings bulging, leaving normal membranous wings (vi); colossal pupae that were larger than normal pupae (vii); and disabled pupae that lacked some body parts (viii) (Fig. 4). All vii and viii pupae died with the abnormal phenotype. Three types of abnormal adults were observed: adults that survived with both sets of elytra slightly opened (ix); adults that partially shed their pupal cuticle with both sets of elytra open and membranous wings that were dislocated, leading to death in all of these individuals (x); and adults that showed combined characteristics of both pupae and adults, which were unable to extricate their appendages and died without completing adult eclosion (xi). All of the ix adults developed from “tiny abnormal” larvae that appeared abnormal in the larval stage and x adults that developed from most of the vi and v larvae; a small number of the iv pupae, vi pupae and v pupae became xi adults, and most of the iv larvae died with a burnt coloration on their body.

### Effects of *TcTre* RNAi on the expression of the TPS, GS and GP genes

*TPS*, *GS* and *GP* are the key genes involved in energy metabolism during insect growth and development. qRT-PCR was used to detect mRNA levels after RNAi targeting the *TPS*, *GS* and two *GP* genes. The expression of *TPS* decreased from 12 to 72 h after *TcTre* RNAi, and its expression was decreased significantly at 12, 36 and 48 h (Fig. 5). In addition, *TPS* mRNA expression decreased significantly during the 24 h after *TcTre1*-*2*, *TcTre1*-*4* and *TcTre2* were knocked down, and its expression decreased significantly during the 72 h after *TcTre1*-*3* and *TcTre2* were silenced.

**Fig. 6:**
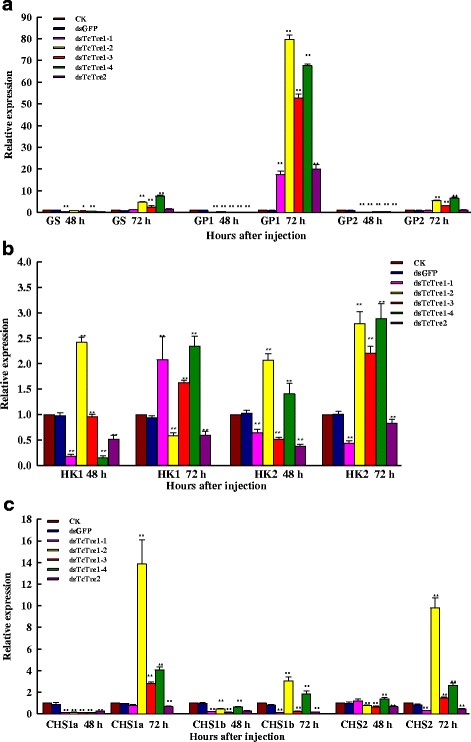
Effects on key genes in the chitin biosynthesis pathway after specific RNAi in last-instar larvae at 48 and 72 h. The levels of these genes relative to the *Tcβ*-*actin* mRNA level were measured via qRT-PCR. **a** mRNA levels of glycogen synthase (*GS*) and glycogen phosphorylase (*GP*). **b** mRNA levels of two hexokinases (*HK1* and *HK2*). **c** mRNA levels of three chitin synthases (*CHS1a*, *CHS1b* and *CHS2*)

The results showed that the expression of the *TPS*, *GS* and the two *GP* genes was decreased significantly at 48 h after dsTcTre injection (Figs. 5 and 6a). The transcript levels of *TPS*, *GS*, *GP1* and *GP2* showed consecutive variation after RNAi targeting the five dsTcTres at 72 h. In addition, *GP1* and *GP2* exhibited high transcript levels at 72 h after dsTcTre1-2, dsTcTre1-3 and dsTcTre1-4 RNAi. The highest transcript levels of *GP1* and *GP2* were 79.6 and 6.6 times those in the respective control group injected with dsGFP (Fig. 6a). Moreover, the ascensional range of *GP1* gene expression was much higher prior to *GP2* gene expression.

### Knockdown of *TcTres* and the expression of genes in the chitin biosynthesis pathway

qRT-PCR was used to detect mRNA levels after RNAi targeting all of the *TcTres*, including eight genes in the chitin biosynthesis pathway (*HK1*, *HK2*, *GPI*, *PFK*, *GFAT*, *CHS1a*, *CHS1b*, *CHS2*). The expression of *HK1* and *HK2* decreased significantly in the 48 h after the injection of the *TcTre1*-*1*, *TcTre1*-*3* and *TcTre2* dsRNAs, whereas their expression increased after dsTcTre1-2 injection (Fig. 6b). The expression of *HK1*, *HK2*, *CHS1a*, *CHS1b* and *CHS2* was decreased substantially after dsTcTre2 RNAi at both 48 and 72 h (Fig. 6b, c). The expression of *CHS1a* and *CHS1b* decreased slightly after RNAi targeting the four soluble dsTcTres at 48 h (Fig. 6c), and the expression of *CHS2* either increased or decreased in the various soluble *TcTre* RNAi groups. The transcript levels of *HK1*, *HK2*, *CHS1a*, *CHS1b* and *CHS2* exhibited consecutive variations after RNAi targeting the five dsTcTres at 72 h. The expression of *HK2*, *CHS1a* and *CHS1b* increased significantly at 72 h after dsTcTre1-2, dsTcTre1-3 and dsTcTre1-4 RNAi (Fig. 6b, c).

**Fig. 7:**
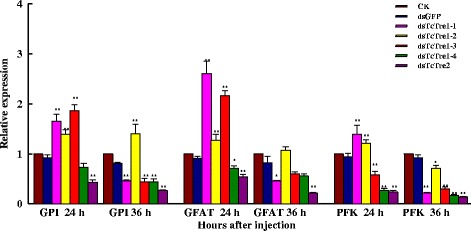
Effects on other genes in the chitin biosynthesis pathway after specific RNAi in last-instar larvae at 24 and 36 h. The mRNA levels of glucose phosphate isomerase (*GPI*), fructose phosphate transaminase (*GFAT*) and phosphofructokinase (*PFK*) relative to the *Tcβ*-*actin* mRNA level were measured via qRT-PCR

The results also showed that *GPI*, *GFAT* and *PFK* gene expression decreased significantly in the 24 and 36 h after TcTre2 gene expression was inhibited through RNAi (Fig. 7). The expression of *GPI*, *GFAT* and *PFK* was increased at 24 h after *TcTre1*-*1*, *TcTre1*-*2* and *TcTre1*-*3* RNAi but was decreased at 24 h after *TcTre1*-*4*, *TcTre2* RNAi, with the exception of a decrease in *PFK* transcript levels after *TcTre1*-*3* RNAi observed at 24 h (Fig. 7). In addition, three gene transcripts were decreased significantly at 36 h after RNAi targeting the five *TcTres*, except that there was an increase after *TcTre1*-*2* RNAi.

## Discussion

RNAi has been extensively studied in insects in recent years and applied in pest control management [7, 37, 38]. In most cases, RNAi results in a low rate of deformed phenotypes in insects and shows varying efficiency among insect tissues [5, 39, 40]. A previous study revealed that dsRNA is more efficient in *T. castaneum*, maintaining the silencing effect for a longer period and inducing a higher rate of deformations [7]. In this study, the dsRNAs for the five *TcTres* were demonstrated to be highly selective and clearly discriminant, and they were verified to be accurate after cloning; the levels of the targeted mRNAs were greatly reduced, along with compensatory expression of the other *TcTres* transcripts after RNAi targeting any of the *TcTres* (Figs. 2 and 3). For example, *TcTre1*-*1* transcripts are reduced not only by dsRNA for this gene but also by dsRNAs for *Tre1*-*2*, *Tre1*-*3* and by *Tre*-*2* genes at 48 h and by dsRNA for *Tre*-*2* gene at 72 h (Fig. 2a). It is clear that there is compensating over-expression of *Tre1*-*3* and *Tre1*-*4* genes at 72 h (Fig. 2a). Similarly the *Tre1*-*2* transcript suppression is not restricted to dsRNA for this gene but also by all other dsRNAs including those of the *Tre*-*2* gene (Fig. 2b). The results indicate that one of *Tre1*-*1*, *Tre1*-*2* and *Tre1*-*3* was inhibited, and the other genes were also lower expression or compensating over-expression, while other Tre transcripts compensating over-expression after *Tre1*-*4* or *Tre2* knockdown and the aberration and mortality rates was more higher than other three *Tre* genes (Table 2). These results are consistent with other observations showing that RNAi is effective in *Tribolium* larvae, pupae and adults, and even in embryos [41, 42].

There are many reports describing the characteristics of the two forms of trehalases. The tissue distribution of the two forms of trehalases differs. Tre1 is mainly expressed in the midgut and Malpighian tubules in *B. mori* and is not expressed in the fat body, silk gland, ovary, trachea, or brain [23, 43–45]. Tre2 is expressed in ovary cells, the spermatophore and the midgut in locusts and in the visceral muscle surrounding the midgut in *B. mori*; Tre2 exhibits both inactive and latent forms that can be activated by being released after destruction of membrane integrity [23, 46, 47]. Zhang also reported that Tre1 is expressed in the midgut, Malpighian tubules and ovary cells, whereas Tre2 is expressed in the fat body, midgut and Malpighian tubules [48]. However, both Tre1 and Tre2 are expressed in the cuticle, midgut, Malpighian tubules, tracheae and fat body of *S. exigua* [28], which is very similar to *T. castaneum*, and all five Tres genes are expressed in the cuticle, fat body, midgut and Malpighian tubules (Fig. 1).

Trehalase participates in both homeostasis and development and is involved in blood sugar metabolism in insects as well as flight metabolism, chitin synthesis during moulting and cold tolerance [26, 27, 49–53]. In addition, Tre2 is expressed as a transmembrane enzyme with an active site on the outside of the cell membrane and mainly hydrolyses extracellular trehalose, whereas Tre1 is located within the cell and hydrolyses intracellular trehalose [44, 54]. Some studies based on injection of the hormone 20-hydroxyecdysone (20E) into *B. mori* have observed an increase in *Tre1* transcript levels and no effect on the expression of *Tre2*, which indicates that insect moults are more closely associated with *Tre1* [44]. Many studies have also shown that soluble *Tre* levels increase before insect moults and after injecting 20-hydroxyecdysone (20E) [19, 28, 44]. In the present study, *GP1* and *GP2* increased rapidly at 72 h (Fig. 5b), as did *HK2* (Fig. 6a), most likely to compensate for the reduced level of the *TcTre1*-*2* transcript, ensuring that the downstream *CHS* gene transcripts increased significantly (Fig. 6c). This result is consistent with the idea that conversion of glycogen into trehalose occurs in last-instar larvae [55]. At the same time, the *CHS1b* transcript decreased to a lower level, although its upstream gene transcripts *GP1*, *GP2*, *HK1* and *HK2* were increased substantially at 72 h after dsTcTre1-3 RNAi in the last-instar larvae (Figs. 5b and 6a, c). These results implied that the *TcTre1*-*3* gene plays a role in energy metabolism by hydrolysing trehalose into glucose and regulating the expression of the *CHS1b* gene, which was demonstrated indirectly based on the 33 % yield of xi adults; the xi adults exhibited a phenotype similar to that resulting from loss of the *CHS1b* gene in *Tribolium* after *CHS1b* RNAi [22]. We found that after dsTcTre1-4 injection, the *GFAT* transcript level was decreased to 0.71-fold of that in the control group injected with dsGFP at 24 h and to 0.55-fold at 36 h, whereas the *PFK* transcript level was decreased more strongly, to 0.27-fold at 24 h and 0.17-fold at 36 h (Fig. 6b). However, 90 % (nine insects) of the iv pupae (a greater percentage than for the other *TcTre* RNAi groups) showed a new phenotype that did not appear in Arakane’s study due to knockdown of the *CHS* genes, which implied that this abnormal phenotype may be due to a lack of energy [9].

In addition, the 40 % abnormal insects and 39 % mortality recorded appeared to demonstrate that *TcTre2* is important, and the uniform distribution of many abnormal phenotypes indicated that *TcTre2* can adjust the expression of other *TcTres* (Fig. 4 and Table 2). All of the *TcTres* in the last-instar larvae were decreased sharply from 12 to 72 h after dsTcTre2 injection compared with the two control groups (Fig. 3); in contrast, *TcTre2* expression increased after RNAi targeting the four soluble dsTcTres, except for *TcTre1*-*2* and *TcTre1*-*4* at 12 h, *TcTre1*-*1* at 36 and 72 h and all of the soluble *TcTres* at 48 h (Fig. 3f). The same phenomenon was observed in the penultimate-instar larvae from 48 to 72 h after dsTcTre2 injection (Fig. 2), and a possible explanation for these findings is that the shortage of the *TcTre2* transcript resulted in a substantial loss of intracellular glucose following the decline of intracellular trehalose. This explanation is consistent with the finding that the expression of *TPS* decreased from 12 to 72 h after dsTcTre2 RNAi (Fig. 5a), and all of the *TcTres* therefore decreased as a corresponding response. These results led to the hypothesis that TcTre2 is a trehalase that mainly hydrolyses extracellular trehalose into glucose flowing into the cell, which is not inconsistent with the idea that Tre2 is a transmembrane enzyme with an active site on the outside of the cell membrane in *B. mori* [44]. Tre2 protein expression was shown to increase substantially, and there was no Tre1 protein present due to the hydrolysis of trehalose into glucose for glycogen accumulation in *B. mori* injected with diapause hormone, which revealed that *Tre2* plays a role in energy metabolism, similar to the finding that *Tre2* targets the hydrolysis of trehalose into glucose that will flow into the oocytes of *B. mori* and *Rhodnius prolixus* [56–58]. In addition, Becker found that the relationship between *GP* and *Tres* in non-feeding insects shows a positive correlation, involving increased expression and activity of *GP* along with a decrease in *Tres*. Our qRT-PCR results revealed that some gene transcripts in both the chitin synthesis pathway and the energy synthesis pathway decreased, including *HK*, *CHS*, *GPI*, *GFAT*, and *PFK*, although *GP1* increased substantially at 72 h and did not sufficiently compensate for the decline in glucose resulting from *TcTre2* RNAi (Figs. 5b and 6). This finding is consistent with Becker’s observation that the insects lacked energy after dsTcTre2 injection [55, 59, 60].

Chitin biosynthesis is well established as a key target for pest control, and trehalase inhibitors are being developed for future pest management. It was reported that inhibition of the expression of the *Laodelphax stritellus* soluble and membrane-bound trehalase genes via the dsRNA feeding method resulted in mortality rates of 38.89 and 27.72 %, respectively [29]. In the pupal stage, the appearance of 60 % v and vi pupae among all of the abnormal pupae may also have resulted from the severe decline in the *CHS1a* transcript and *CHS1b* transcript, which caused a higher death rate (12 %) in the adult stage compared with the other *TcTre* RNAi groups (Fig. 4b). The abnormal pupal and adult phenotypes were consistent with Arakane’s findings that abnormal iii larvae and adults emerged after RNAi targeting *CHS1a*, and vi adults emerged after RNAi targeting *CHS1b* or other genes [9, 61, 62].

As it known that the same family genes which have relative high protein sequence identity, so the compensatory function or cross silencing have been found in gene function study by RNAi. In the study of *Nilaparvata lugens TPS* genes function, *NLTPS2* gene’s expression followed decreased when *NLTPS1* gene’s expression inhibited by RNAi [63]. In our study, the expression of *TcTre1*-*1* also decreased when dsTcTre1-1, dsTcTre1-2, dsTcTre1-4 and dsTcTre2 were injected from 12 to 36 h, but it increased when dsTcTre1-3 injected from 24 to 72 h (Fig. 3a). As well as *TcTre1*-*2* (Fig. 3b), *TcTre1*-*4* (Fig. 3d) and *TcTre2* (Fig. 3e) have compensatory function at some extent when other *TcTre* was knockdown. The seeming cross silences similar with *TPS* genes function study also been found [63], while it is also reported that *N. lugens* soluble trehalases (*NlTre1*-*1* and *NlTre1*-*2*) and membrane-bound trehalase (*NlTre2*) also have the compensatory function [64]. The good inhibited effects and stronger phenotype were found when two *Tre1* and one *Tre2* gene in *N. lugens* was knockdown simultaneously [64]. However, double knockdown *vti1a* and *vtilb* did not produce a strong phenotype and it suggesting a compensatory mechanism exists within the secretory system though v*ti1a* and *vti1b* by RNAi resulted in a significant decrease in the study of Vti family of SNARE proteins function [65]. Therefore, the crossing silencing and compensatory mechanism of the same gene family need more studied in the future.

## Conclusions

These results demonstrate that the five trehalase genes exhibit different functions in chitin biosynthesis and energy metabolism by the way of RNAi. In particular, a mortality rate close to 40 % resulted from the silencing of TcTre1-4 or TcTre2 gene expression through RNAi, and it could be effective for pest control by a potential trehalase inhibitor [66]. Moreover, the development of anti-trehalase agents that target the chitin biosynthesis pathway or energy metabolism is an alternative method for exploring potential chemical insecticides.

## Additional files

Additional file 1: Figure S1. Synthesis and degradation of trehalose and glycogen in insects. Adapted from Rockstein [16], Elbein et al. [17], Montooth et al. [18], Kunieda et al. [19], and Tang et al. [20]. The red arrow and dotted arrow indicate that the enzyme in this pathway was analysed and that this pathway is unknown, respectively. **Figure S2:** Alignment of the nucleotide sequences of five TcTre genes in the region of the dsRNAs. (DOCX 21 kb)

## Abbreviations

cDNA: Complementary DNA
CHS: Chitin synthase
CK: Control group
Cu: Cuticle
DMRT: Duncan’s new multiple range test
dsGFP: Double-stranded GFP RNA
dsRNA: Double-stranded RNA
dsTcTre: Double-stranded trehalase RNA of *Tribolium castaneum*
Fb: Fat body
GFAT: Glutamine, fructose-6-phosphate transaminase
GFP: Green fluorescent protein
GP: Glycogen phosphorylase
GPI: Glucose-6-phosphate isomerise
GS: Glycogen synthase
h: Hour
HK: Hexokinase
Mg: Midgut
Mt: Malpighian tubules
PCR: Polymerase chain reaction
PFK: Phosphofructokinase
PM: Peritrophic membrane
qRT-PCR: Quantitative real-time polymerase chain reaction
RNAi: RNA interference
RT-PCR: Reverse transcription polymerase chain reaction
SEs: Standard errors
Tc: *Tribolium castaneum*
TPS: Trehalose-6-phosphate synthase
Tre: Trehalase
μg: Microgramme
μl: Microliter
