# Supplementary material for: Knockdown of five trehalase genes using RNA interference regulates the gene expression of the chitin biosynthesis pathway in Tribolium castaneum
Source: BMC Biotechnol. 2016 Sep 6;16(1):67. doi: 10.1186/s12896-016-0297-2 (PMC5011928; doi:10.1186/s12896-016-0297-2)
Supplement: Additional file 1: Figure S1. — Synthesis and degradation of trehalose and glycogen in insects. Adapted from Rockstein [16], Elbein et al. [17], Montooth et al. [18], Kunieda et al. [19], and Tang et al. [20]. The red arrow and dotted arrow indicate that the enzyme in this pathway was analysed and that this pathway is unknown, respectively. Figure S2: Alignment of the nucleotide sequences of five TcTre genes in the region of the dsRNAs. (DOCX 21 kb) [file 12896_2016_297_MOESM1_ESM.docx]

**Figure S1** Synthesis and degradation of trehalose and glycogen in insects. Adapted from Rockstein *et al*. (1978), Elbein *et al*. (2003), Montooth *et al*. (2003), Kunieda *et al*. (2006), and Tang *et al*. (2012). The red arrow and dotted arrow indicate that the enzyme in this pathway was analysed and that this pathway is unknown, respectively.

Trehalose

β-D-Glucose

Glucose-6-phosphate

Hexokinase (HK)

Trehalose-6-phosphate Synthase (TPS)

Glycogen phosphorylase (GP)

Chitin Biosynthesis Pathway

Glycolysis Pathway

Molting, metamorphosis or developmental process delays

Fructose-6-phosphate

GPI (glucose phosphate isomerase)

GFAT (frutcose phosphate transaminase)

PFK (phosphofructokinase )

Glycogen

Trehalose-6-P

Trehalose-6-phosphate phosphatase (TPP)

Trehalase （Treh1 and Treh2）

Glycogen synthase (GS)

**Figure S2** Alignment of the nucleotide sequences of five *TcTre* genes in the region of the dsRNAs.

1 70

TcTre1-1 (1) ---------CGACCTGAAATTAGCCCAGAAGCGGAAAAACTTCTACCCCA--GTAATTTGACCCCTTTGT

TcTre1-2 (1) --------ACGATATCGAGCTCAGAGTCCAAAGAAAACATTTCTATCCCA--GCAACTTTGCGCCGCTCT

TcTre1-3 (1) --------GGGACCGTGGGTTAAACCAACCACGGAAGGGTTACTACGCCA--GTAATTTAACCCCACTGT

TcTre1-4 (1) ---------GGACAATGAGTTGGGGCAGCACCGCAAGTACTTCTTCCCTT--CTAATTTGGCCCCCTTGT

TcTre2 (1) CTACAAAGCGTTCAAAGAGACCGACGCCATGTTTGAAAAGTACGACGCGACGGTGCCTGGGGGCCACGGC

71 140

TcTre1-1 (60) GGGCGCGCGC-GAGCCACGAAATCGACGCGG-------GGAAAATCGTGC-----AGTA--TC-TGGGGC

TcTre1-2 (61) GGACTGAGTG-TTACGACACCAGCATCAGGCAAAGTTACGGGAAAAACGCCACCAATTATCTGGTCCAGA

TcTre1-3 (61) GGACACAATG-CTACGACCCCAACCTTTCGGATCATTTGGGCCAGAAAGCAGTCCAGTACTTGTCAAAAA

TcTre1-4 (60) GGGCGGAGGC-GTACGACTTGTCCAAGGCCGATGTTCTGGGCCAGCGGGCGGCTGAGTACGTCGTGAGGC

*TcTre2* (71) GGGGGCGGCGAGTACGAGACGCAGCTAGGGT-TCGGTTGGACGAACGGGATCATCATGGATTTGTTGTAC

141 210

TcTre1-1 (114) A---GGTTTTGGGGTACGGGGGCGGCGTGCCTGCGTCCTTGACCAAGTCGGGGCAGCAGTGGGACTTCCC

TcTre1-2 (130) AGGGGATTCTCAACTACGAAGGGGGGATTCCCACGTCCCTGTTTGAGACAGGGGAACAGTGGGACATGCC

TcTre1-3 (130) CCGGGATTCTGGACTTTGATGGTGGCATCCCTGCCTCCCTTGTGAACAGTGGGGAGCAATGGGACTTCCC

TcTre1-4 (129) AAAAACTGCTGGACTACCAAGGGGGCATTCCAGCCTCCTTGACCAGGAGCGGGGAGCAGTGGGACTACCC

TcTre2 (140) CGATACTCGGGGAACCTGACTGTGGAGGACCCGCCACCACCGTCACCAAAACCGGTCTTTGAGGCGTCGC

211 280

TcTre1-1 (181) GGGGGCTTGGCCCCCTTTGCAGGAACTTGTCATAAATTCGCTCTACC-GTACGAAGGATTCCAAAGCGAT

TcTre1-2 (200) AAATGCGTGGGCCCCCACACAGGCAATTGTGATTTTTGGGCTGGATA-AGAGCCAGGATCCTGGAGCGAA

TcTre1-3 (200) CAACGCCTGGCCGCCCCTCCAGTCAATCGTCATTCTGGGCTTGGACC-ACACAGGCCACCCCCAAGCCCA

TcTre1-4 (199) CAACGCCTGGCCCCCGCTGCAGAGCCTCGTGGTCATGGGTCTGGACA-GGAGCGGCAACTGCAAGGCCAA

TcTre2 (210) -AAGACGTGCAATCTGCGTCGAGTTTTAGCCAAATTTCGGCCGTCTTGATAGCGTTAATGATATCGCTAA

281 350

TcTre1-1 (250) --TGAGGTGGCTAAGGAGT-TGTCGCAAAAATGGCTCAAGTCTAATGTCCAGGCGTTCGATAAATATGGG

TcTre1-2 (269) --GAAGGTGGCCCAGGACT-TGGCGTTTCGCTGGATTGATTCGTTGGTTAAAGTGGCAGAAGACACCCAT

TcTre1-3 (269) --GAAAACCGCCCAAGATT-TGGCCGAGAAGTGGATTAGGTCAAATTTGGACAGTTTCAAGGCCACGGGC

TcTre1-4 (268) --GGAGCTGGCCCGGGAGT-TTGCCCAACGATGGGTCACTGCCAATTTGATTGGGTTCAACCAGACCAGC

TcTre2 (279) CGGCAGGATTCATAGGAGTGTGTATCTACAAACGCCGGAACCAGCAGCAGACCGACTCGAGGAAAGCGCC

351 420

TcTre1-1 (317) GCCATGTTTGAGAAGTATG--ACGCTGAAAGAGTGGGGGAGCC---------------------------

TcTre1-2 (336) GAGATGTTCGAGAAGTACA--ACGCCATGTTCAAGGGGATGTATGGGGGTGGGGGCGAGTATGAGGTCCA

TcTre1-3 (336) CAAATTTCCGAGAAGTACG--ACGTTCAGTTCAGCGGCCACAG---------------------------

TcTre1-4 (335) GAAATGTTCGAGAAGTACG--ACGCGGAGGTGCCGGGACAGTATGGAGGGGGCGGGGAGTACGTCATCCA

TcTre2 (349) TAGGCCTTCAAGG-GCACGCTACACGGAACTGCGGAGCAT------------------------------
